# Amygdala hyper-connectivity in a mouse model of unpredictable early life stress

**DOI:** 10.1038/s41398-018-0092-z

**Published:** 2018-02-21

**Authors:** Frances K. Johnson, Jean-Christophe Delpech, Garth J. Thompson, Lan Wei, Jin Hao, Peter Herman, Fahmeed Hyder, Arie Kaffman

**Affiliations:** 1 Department of Psychiatry, Yale University School of Medicine, 300 George Street, Suite 901, New Haven, CT 06511 USA; 2 Department of Newborn Medicine, Boston Children’s Hospital, Harvard medical school, Boston, MA 02115 USA; 3 Department of Radiology & Biomedical Imaging and Magnetic Resonance Research Center, Yale University, New Haven, CT 06520 USA; 4 iHuman Institute, ShanghaiTech University, 393 Middle Huaxia Road, Ren Building, Room B204, Zhangjiang, Pudong, Shanghai 201210 China; 5 Department of Biomedical Engineering, Yale University, New Haven, CT 06519 USA

## Abstract

Childhood maltreatment is associated with a wide range of psychopathologies including anxiety that emerge in childhood and in many cases persist in adulthood. Increased amygdala activation in response to threat and abnormal amygdala connectivity with frontolimbic brain regions, such as the hippocampus and the prefrontal cortex, are some of the most consistent findings seen in individuals exposed to childhood maltreatment. The underlying mechanisms responsible for these changes are difficult to study in humans but can be elucidated using animal models of early-life stress. Such studies are especially powerful in the mouse where precise control of the genetic background and the stress paradigm can be coupled with resting-state fMRI (rsfMRI) to map abnormal connectivity in circuits that regulate anxiety. To address this issue we first compared the effects of two models of early-life stress, limited bedding (LB) and unpredictable postnatal stress (UPS), on anxiety-like behavior in juvenile and adult mice. We found that UPS, but not LB, causes a robust increase in anxiety in juvenile and adult male mice. Next, we used rsfMRI to compare frontolimbic connectivity in control and UPS adult male mice. We found increased amygdala–prefrontal cortex and amygdala–hippocampus connectivity in UPS. The strength of the amygdala–hippocampal and amygdala–prefrontal cortex connectivity was highly correlated with anxiety-like behavior in the open-field test and elevated plus maze. These findings are the first to link hyperconnectivity in frontolimbic circuits and increased anxiety in a mouse model of early-life stress, allowing for more mechanistic understanding of parallel findings in humans.

## Introduction

Childhood maltreatment in the form of abuse, neglect, or erratic parenting is responsible for almost half of all childhood psychiatric disorders in the USA including anxiety, depression, and behavioral dysregulation^1–3^. In the absence of effective interventions, childhood psychopathologies often progress to chronic psychiatric and medical conditions in adulthood^2,4,5^. Work from the Adverse Childhood Experiences (ACE) study found a robust dose-dependent relationship between the number of adverse events early in life and the risk for multiple psychopathologies in adulthood including anxiety, depression, psychosis, somatization disorders, and substance abuse^6^. It is currently unclear how multiple adverse events early in life synergize to affect such a broad clinical presentation and whether different types of childhood maltreatment (e.g., sexual abuse, physical abuse, emotional neglect, and emotional abuse) lead to different developmental and behavioral consequences^2,7^. Moreover, conflicting findings are found in the literature regarding the ability of sex to modify the consequences of early-life stress (ELS), with some reports suggesting that childhood adversity causes different outcomes in men and women^8–11^ while others find similar outcomes regardless of sex^12–14^.

A major obstacle in clarifying some of these clinical observations is the over reliance on subjective reports about sensitive and personal information usually obtained by a complete stranger^8,9,15^. In this regard, the recent use of functional imaging provides an important and promising objective tool to study the effects of childhood adversity on brain function and connectivity. Indeed, work from several groups has consistently found increased activation of the amygdala in response to threat in individuals exposed to childhood maltreatment^16–24^ and similar findings have been replicated in animal models of ELS^17,25^. The results with resting-state fMRI (rsfMRI) are mixed with some groups reporting reduced connectivity between the amygdala and other limbic structures such as the prefrontal cortex and the hippocampus^26–29^, while others have found either no effect or increased connectivity in individuals with childhood maltreatment^30–32^. These conflicting results are likely due to differences in the form and the severity of childhood maltreatment, the sex and age of the subjects, and the presence of additional comorbidities (summarized in Table S1).

Exposure to stress early in life leads to robust behavioral changes in a wide variety of species including rodents and nonhuman primates^4,33,34^. These changes include: increased anxiety, altered response to threat, abnormal hippocampal-dependent memory, and increased hypothalamic-pituitary-adrenal (HPA) reactivity^4,33,34^. Recent advances in imaging have allowed the use of rsfMRI to characterize connectivity in small rodents^35^. The utility of these studies is particularly powerful in the mouse where the genetic background and the ELS paradigm can be rigorously controlled. In addition, alterations in connectivity found by rsfMRI can be further characterized using anterograde and retrograde labeling techniques, electrophysiology, and optogenetics. However, no group has yet used rsfMRI to characterize connectivity in a mouse model of ELS.

To address this issue we first compared the effects of two paradigms of ELS on anxiety-like behavior in juvenile and adult Balb/cByj mice, a strain that was chosen because of its sensitivity to other paradigms of ELS^36–40^. The first paradigm is a modified limited bedding (LB) protocol^41^ in which pups are raised in the presence of limited bedding and no nesting material from postnatal day (P0) to P25. Exposure to LB has been shown to cause fragmentation of postnatal maternal care and elevated corticosterone levels during the postnatal period and adulthood^41^. Adult mice exposed to LB show increased HPA reactivity, impaired hippocampal-dependent learning, but mixed effects on anxiety-like behavior^33^. In the second paradigm, which we named unpredictable postnatal stress (UPS), pups are raised with limited bedding in the same way described for the LB condition. However, in contrast to LB pups that are left undisturbed, UPS pups are also separated for 1 h from their dam using an unpredictable schedule (e.g., P14, P16, P17, P21, P22, and P25) and the nest in their home cage is disrupted (see Methods section for details). We hypothesized that the unpredictability and complexity of the UPS paradigm during a developmental period when the HPA is fully matured in mice^37,42,43^ would mimic the dose-dependent effect seen in children exposed to multiple adverse outcomes early in life^6^ and would lead to a more robust anxiety-like phenotype in UPS compared to LB and control offspring. In addition, we postulated that rsfMRI would reveal altered frontolimbic connectivity between the amygdala, prefrontal cortex, and the hippocampus in adult mice exposed to UPS.

## Methods

### Animals

BALB/cByj mice (Stock # 001026, Jackson Laboratories) were housed in standard Plexiglas cages and kept on a standard 12:12 h light–dark cycle (lights on at 0700 hours), constant temperature and humidity (22 °C and 50%) with food provided ad libitum. All studies were approved by the Institutional Animal Care and Use Committee (IACUC) at Yale University and were conducted in accordance with the recommendations of the NIH Guide for the Care and the Use of Laboratory Animals.

### Early-life stress models

**Fig. 1:**
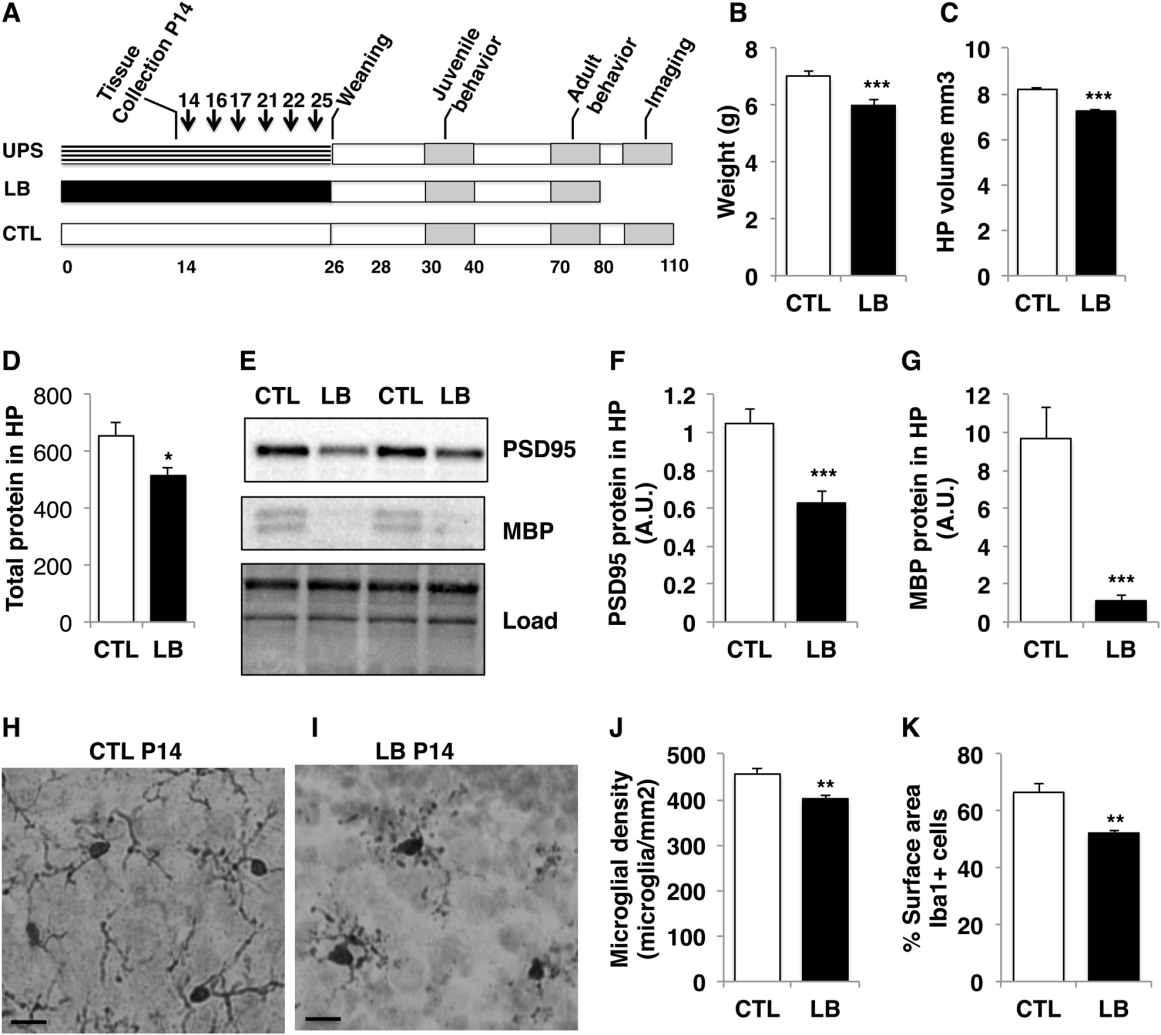
Exposure to LB perturbs normal hippocampal development in 14-day-old pups. (**a**) Experimental design and timeline. (**b**) Body weight at P14, CTL = 25, LB = 42. (**c**) Hippocampus volume, CTL = 5, LB = 5. (**d**) Total protein extracted from the left hippocampus. (**e**) Western blot showing levels of PSD95 and MBP protein levels in the hippocampus of 14-day old male pups. Quantification of PSD95 (**f**) and MBP levels (**g**), *A.U.* arbitrary units, CTL = 7, LB = 7 (**d**–**g**). Representative pictures of Iba1-positive microglia in the hippocampus of P14 CTL (**h**) and LB P14 male pups (**i**). Exposure to LB reduces the density (**j**) and the surface area (**k**) of Iba1-positive cells in the developing hippocampus, CTL = 5, LB = 5 (**j**–**k**). Error bars represent mean ± s.e.m. *p < 0.05, **p < 0.01, ***p < 0.0005 independent *t*-tests. Scale bars in H & I are 10 μm

Sixty female and 30 male BALB/cByj mice, 7 weeks old, were purchased from the Jackson Laboratory and allowed to acclimate for 20 days in our animal facility. Mice were then mated using 3:1 females to male harems in standard mouse Plexiglas cages layered with 500 cm^3^ corncob bedding but with no nesting material. Visibly pregnant dams were transferred to ‘maternity cages’ containing 500 cm^3^ corncob bedding but no nesting material. At birth, postnatal day (P0) litters were culled to 6–8 pups and randomized to either control (CTL), limited bedding (LB), or unpredictable postnatal stress (UPS) conditions. Mice raised under CTL condition were provided with 500 cm^3^ corncob bedding and one 5 × 5 cm nestlet per cage. Bedding for CTL condition was changed on P14 and P21. LB and UPS litters were provided with 125 cm^3^ of corncob and no nesting material from P0–25 with bedding changes on P7, P14, and P21. UPS litters were also separated from their dam for 1 h on P14, P16, P17, P21, P22, and P25. During the separation period the dam was transferred to a new cage followed by the individual transfer of the pups to a different cage containing clean corncob bedding. During the separation period, the home cage was briefly shaken to evenly spread the bedding and disrupt the nest. At the end of the 1 h separation, the pups were returned to their home cage followed by the return of the dam. At P26, mice were weighed, sexed, and housed in a cage containing 500 cm^3^ corncob and no nesting material with 3–5 other mice of the same sex and experimental condition (Fig. 1a).

### Western blot

Mice were rapidly decapitated and the hippocampi were dissected rapidly and frozen in liquid nitrogen. All dissections were done between 1400–1700 hours to minimize circadian effects on gene expression. The left hippocampus was rapidly thawed in cold lysis buffer from the AllPrep DNA/RNA/Protein mini kit (Cat# 80004, Qiagen), homogenized (10 × 1 s pulses) using an ultrasonic processor (Cole Parmer CP70), and spun at 16 000 × *g* for 3 min. RNA, DNA and proteins were then purified using the AllPrep DNA/RNA/Protein mini-kit according to manufacturer’s instructions. Western blots were done and quantified as described previously^40^. Briefly, protein samples (50 μg/lane) were separated on a 12% Criterion TXG Stain Free midi-gel (cat# 567–8044, BioRad), transferred to a nitrocellulose membrane (Cat#170–4159, Bio-Rad), and incubated with mouse anti-PSD95 antibodies (Millipore, Cat# MAB1598, 1:1000) and rabbit anti-MBP antibodies (Abcam, Cat# ab2404, 1:500). Proteins were detected using Western Lightning Western Blot Chemiluminescence Reagent Plus kit (NEL104001EA, PerkinElmer), quantified using the ChemiDoc XRST imaging system, and normalized to membrane load^44^.

### Cell counting and stereological analyses

Male pups (P14) were anesthetized with chloral hydrate (100 mg/kg) and transcardially perfused with cold PBS/heparin (50 u/ml) solution followed by 10% formalin (polyScience). Brains were then post-fixed overnight at 4** °**C **i**n 10% formalin and then equilibrated in 30% sucrose solution. Forty-micron coronal sections were collected in 6 pools, each containing 16–18 slices spaced at 240-micron intervals that systematically span the entire rostral-caudal axis of the hippocampus. One pool of slices was incubated overnight with rabbit anti Iba1 (Wako, Cat. #019–19741, 1:250) followed by biotinylated goat anti-rabbit antibodies (Vector Labs,1:250) and visualized using ABC kit (Vector Labs) and 3′,3′diaminobenzidine (DAB) staining. Slices were then dried overnight and counterstained with Hematoxylin stain (Vector Labs). Unbiased stereological counting of Iba1-positive cells in the hippocampus was done using a modified version of the optical fractionator as was previously described^39^. Briefly, the hippocampus borders were delineated under low magnification (×2.5) and the Stereo Investigator 10 software (MBF Bioscience) was used to determine the surface area and to count cells. All counting was done under ×40 magnification by an observer who was blind to the developmental history of the mouse. The total number of Iba1-positive cells in the hippocampus was calculated as *N*(_T_) = Σ*Q*×1/ssf×1/asf×1/tsf, where Σ*Q* (the total number of Iba1 cells counted, *n* = 500–1000 per mouse), ssf (the sampling fraction) = 1/6,asf = (area of counting frame/area of grid) = 1/9, and tsf (thickness sampling fraction) = 1. The hippocampal volume was determined by the Cavalieri’s principle using the Stereo Investigator software^39,45^ and the density of microglia was calculated by dividing the total number of Iba1-positive cells counted (Σ*Q*) by the total surface area of the hippocampus (in mm^2^). To assess Iba1-positive surface area, slices were coded to mask the developmental history of the mouse, and high-resolution digital images of DAB-stained slices were obtained under identical exposure conditions (Zeiss Axioplan, ×10 magnification). The dorsal hippocampus borders were traced and the images were analyzed with imageJ software (NIH) using the automated threshold function as described previously^39^.

### Behavioral testing

Anxiety-like behavior was tested using the open-field test and the elevated plus maze^37,39^. In the open-field test, mice were allowed to explore a 50 × 50 cm arena for 5 min during which the distance traveled and the time spent in the inner 15 cm-area were measured using the EthoVision tracking system (Noldus Information Technology). For the elevated plus maze (EPM), the mice were placed in the middle of a standard elevated plus maze (each arm is 10 × 50 cm long) facing an open arm and allowed to explore the maze for 5 min. The time spent exploring the open and closed arms, the number of entrances, and the distance travelled were determined using the EthoVision tracking system. Anxiety-like behaviors were tested in juvenile (P35–45, CTL = 25–26, LB = 21, UPS = 22–25) and adult mice (P70–80, CTL = 24–26, LB = 21–24, UPS = 19–20) using two independent cohorts (see Fig. 1a for timeline).

### RsfMRI image acquisition, processing, and analysis

At the completion of the behavioral testing, adult male control and UPS mice were transferred to the Imaging Center at Yale where they were housed for 1–2 weeks prior to being imaged. Five male control mice (14–16 weeks old, 25–32 g) and 6 male UPS mice (12–15 weeks old, 23–28 g) were anesthetized with 25% urethane dissolved in distilled water (Sigma Cat # U2500–250G) using intraperitoneal bolus injections (1.7 g/kg) given in three separate dosages. Urethane limits experimental stress and motion during imaging and is thought to produce similar physiology to unanesthetized animals^46^. After the initial sedation step, an intraperitoneal line was placed and additional urethane was administered at 0.1 g/kg dosages until toe pinch reflex was completely suppressed (see Table S3 for the total urethane dosages used). Fomblin Y (Sigma-Aldrich) was placed in ear canals to reduce susceptibility artifacts and Puralube Vet Ointment (Dechra Vet Products) was applied over the ocular surface to prevent desiccation. Anesthetized mice were positioned and fixed with ear/bite bars in a customized cradle for imaging. Body temperature was monitored with a rectal probe and two subcutaneous carbon wires were placed to capture heart and breath rate. Mice were allowed to breathe a mixture of 50% oxygen and 50% compressed air during setup and scanning. Imaging was performed on a 9.4 Tesla MRI scanner (Bruker) with a 12 mm custom-built surface coil. Mice were warmed with a waterbed inside the scanner and body temperature was maintained between 36 and 38 °C. Heart and respiratory rates were monitored throughout. BOLD resting-state fMRI was acquired four times in each animal with echo planar imaging in 8–1 mm-thick coronal slices, across a 12.8 mm × 12.8 mm field of view, in a 32 × 32 matrix, with 1000 ms TR, 13 ms TE, acquiring 384 volumes in 6.4 min. Coplanar anatomical reference images were acquired by a gradient echo sequence in 8–1 mm-thick slices across a 12.8 mm × 12.8 mm field of view, in a 64 × 64 matrix, with 1000 ms TR and 4 ms TE. Image preprocessing and analysis were done in MATLAB (Mathworks, R2016a). Motion correction and slice timing of the functional time series data were done with SPM12 (Wellcome Trust Centre for Neuroimaging, London, UK). Time series data were spatially smoothed with a (6.4 mm, 1.6 mm sigma) Gaussian kernel and bandpass filtered from 0.01 to 0.3 Hz^47^. Data are reported without global signal regression due to concerns of introducing spurious negative correlations and the removal of state-specific information^48–51^. Whole-brain masks and region of interest masks, including the amygdala, prefrontal cortex, ventral hippocampus, dorsal hippocampus, and the hypothalamus were drawn bilaterally using the Allen Mouse Brain Atlas^52^ by a researcher who was blind to the mouse’s developmental history. These selected ROIs constitute 10 nodes with 45 unique node-pair connections. Pearson’s functional correlations for each connection were calculated in individual space and normalized to a *z* score with the Fisher *r* to *z* transformation^53^. Data from four trials were averaged for each mouse to obtain an individual correlation coefficient that was used to calculate the group’s average. Differences in group-averages were evaluated using independent-sample *t*-tests, with *p* < 0.05 (two tails) considered significant. Sequential goodness of fit (SGof) was used to correct for multiple comparisons^54^.

### Statistical analyses

Data were screened for errors, outliers, normality and homogeneity of variance using SPSS Statistics 24 (IBM). Animals or samples that were >2 s.d. above or below the mean and violated normality were eliminated. Independent Student *t*-tests were used to assess the effects of LB on protein expression in the developing hippocampus. A 3 × 2 ANOVA was used to assess the effects of ELS (CTL vs. LB vs. UPS) and sex (males vs. females) on weight and anxiety-like behavior in juvenile and adult mice. This analysis was followed by one-way ANOVA when appropriate (see Results section for details). Significant main effects and interaction were followed by Tukey-HSD post hoc analyses. Bivariate Pearson correlation was used to calculate the correlation between rsfMRI connectivity and anxiety-like behavior. Sample sizes for behavioral testing were based on a 2 × 3 factorial design, with preliminary work showing an estimated effect size of 1.2, *α* = 0.05, and power >0.8. SPSS Statistics 24 (IBM) was used for statistical analysis with *p* < 0.05 (two tails) considered as significant.

## Results

### Exposure to LB causes a developmental delay and long-term reduction in body weight and PSD95 levels in the hippocampus

We first characterized the effects of LB on pup development at P14, prior to the initiation of UPS (Fig. 1a for timeline). Exposure to LB was associated with a 15% reduction in body weight (Fig. 1b) with a 2 × 2 ANOVA showing highly significant effect of LB (*F*[1,63] = 14.9, *p* < 0.0005), with no effect of sex (*F*[1,63] = 0.18, *p* = 0.67), or interaction (*F*[1,63] = 0.43, *p* = 0.52). Next we assessed the effects of LB on hippocampal volume, total protein content, and levels of the synaptic proteins PSD95, mGluR5 and the myelination marker MBP. The volume of the hippocampus (*t*[8] = 9.18, *p* < 0.0005, Fig. 1c) and the total amount of proteins extracted from the hippocampus (*t*[12] = 2.58, *p* = 0.024, Fig. 1d) were both significantly reduced in P14 LB pups, but these results were no longer significant when normalized to body weight (*t*[12] = 0.34, *p* = 0.730). Nevertheless, protein levels of PSD95 were significantly reduced (*t*[14] = 4.38, *p* = 0.001) even after normalization to the total amount of protein loaded (Fig. 1e, f) with a similar trend seen for mGluR5 (*t*[14] = 2.00 *p* = 0.065, data not shown). Levels of the myelination marker MBP were 10-fold lower in LB (*t*[14] = 5.13, *p* < 0.0005, Fig. 1e, g).

Given the sensitivity of microglia to stress and their role in regulating synaptogenesis and myelination^55^ we tested the effects of LB on microglial density and morphology in the hippocampus of 14-day-old pups. Staining tissue with Iba1 antibodies showed that exposure to LB reduced the number of microglia in the developing hippocampus. Microglia from LB pups also showed distinct morphological changes and reduced complexity (Fig. 1h, i). Unbiased cell counting confirmed reduced microglial density (*t*[8] = 3.73, *p* = 0.006, Fig. 1j) and automated image analysis showed significant reduction in the Iba1 surface area of microglia present in the developing hippocampus of LB mice (*t*[8] = 4.37, *p* = 0.002, Fig. 1k). These findings document the presence of multiple developmental abnormalities in 14-day-old LB pups prior to the onset of the unpredictable stress associated with the UPS paradigm.

Next we characterized the effects of LB and UPS on body weight in juvenile (P28) and young adult mice (P60–70). A 3 × 2 ANOVA found a significant effects of ELS (*F*[2167] = 73.95, *p* < 0.0005) and sex (*F*[1167] = 4.11,*p* = 0.044) but no significant interaction (*F*[2167] = 0.15, *p* = 0.86) on body weight at P28. Exposure to LB and UPS caused 15% reduction in body weight that was highly significant in both males and females (Fig S1 A-B), with similar results seen in young adult mice (Fig S1 C-D). There were no significant differences in body weights between LB and UPS mice indicating that exposure to UPS did not exacerbate the long-term effects of LB on growth (Fig S1).

### Effects of LB and UPS on anxiety-like behavior in juvenile mice

**Fig. 2:**
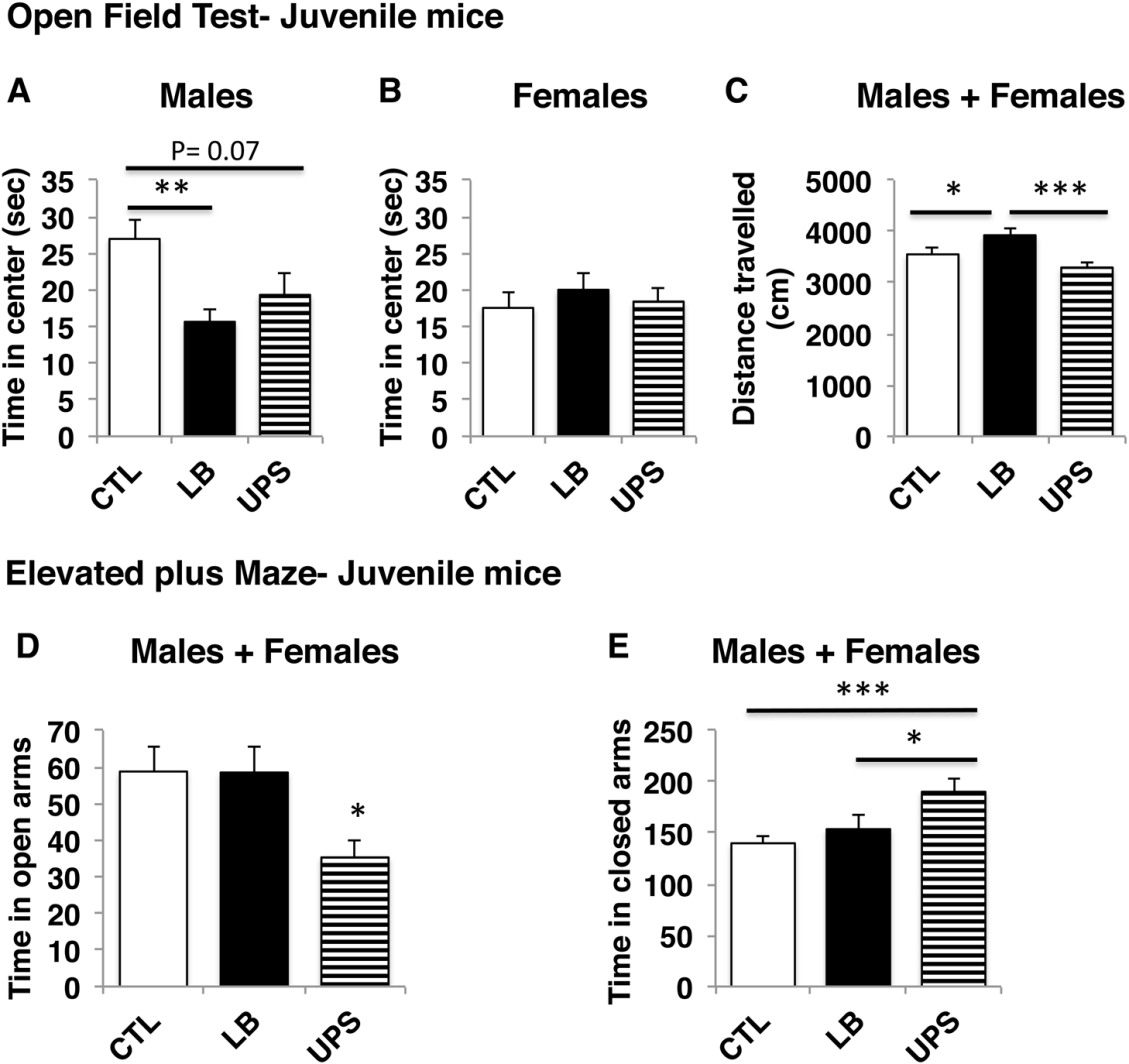
Anxiety-like behavior in juvenile mice (P35–40). Open-field test (**a**–**c**). Exposure to LB and UPS reduced time spent in the center of the open field in juvenile males (**a**) but not female mice (**b**). LB male and female juvenile mice showed increased locomotor activity compared to CTL and UPS mice (**c**). Elevated plus maze (**d**, **e**). UPS male and female mice spent less time in the open arms (**d**) and more time in the closed arms (**e**) compared to LB and CTL mice. Males: CTL = 12, LB = 12, UPS = 10–11, Females CTL = 13–14, LB = 9, UPS = 12–14. Error bars represent mean ± s.e.m. *p < 0.05, **p < 0.001, ***p < 0.0005, Tukey-HSD

Based on the observations that childhood maltreatment leads to an increase in childhood anxiety^1,6,56^, we first compared the effects of LB and UPS on behavior in the open-field test (Fig. 2a−c) and the EPM (Fig. 2d, e) in juvenile mice (P35–40). A 3 × 2 ANOVA found significant interaction between ELS and sex for time exploring the center of the open field (*F*[2,66] = 4.81, *p* = 0.01), with no significant effects of ELS (*F*[2,66] = 2.17, *p* = 0.122) or sex (*F*[1,66] = 1.018, *p* = 0.28). A separate analysis was therefore conducted for males and females. One-way ANOVA found a significant effect of ELS on time exploring the center in males (*F*[2,32] = 6.07, *p* = 0.006) with post hoc analysis revealing a significant reduction in LB (*p* = 0.005) and a trend for reduced time spent in the center for UPS (*p* = 0.07) when compared to CTL males (Fig. 2a, and Table S2 for a summary of the behavioral testing). There was no difference in the time spent in the center of the open field between LB and UPS males. Similar analysis in females showed no effect of ELS on time spent in center of the open field (*F*[2,34] = 0.319, *p* = 0.73, Fig. 2b and Table S2). A 3 × 2 ANOVA for the distance travelled in the open-field found significant effect of ELS (F[2,66] = 8.33, *p* = 0.001) with no significant effect of sex or interaction (Table S2). Post hoc analysis, using both male and females, found increased locomotor activity in LB compared to CTL (*p* = 0.05) and UPS (*p* < 0.0005), with no difference between UPS and CTL (*p* = 0.2, Fig. 1c). These findings suggest that both LB and UPS cause sex-specific increase in anxiety-like behavior in juvenile male mice. In addition, LB but not UPS juvenile mice show increased locomotor activity when exploring a novel arena.

Using the same juvenile mice, we then compared the effects of LB and UPS on exploration in the EPM. There was a significant main effect of ELS on time spent in the open arms (*F*[2,62] = 4.53, *p* = 0.015), with no significant effect of sex or interaction (Fig. 2d and Table S2). Post hoc analysis indicated that UPS mice spent significantly less time in the open arms compared to CTL (*p* = 0.026) and LB (*p* = 0.037), with no difference between LB and CTL. There was also a significant effect of ELS on time spent in the close arms (*F*[2,62] = 8.21, *p* = 0.001), with no significant effects of sex or interaction (Fig. 2e, Table S2). The main effect of ELS was due to increased time spent in the closed arms in the UPS group compared to CTL (*p* < 0.0005) and LB (*p* = 0.015), with no difference between LB and CTL. These findings indicate that juvenile UPS but not juvenile LB mice display increased anxiety-like behavior in the EPM.

### UPS but not LB increases anxiety in adult male mice

**Fig. 3:**
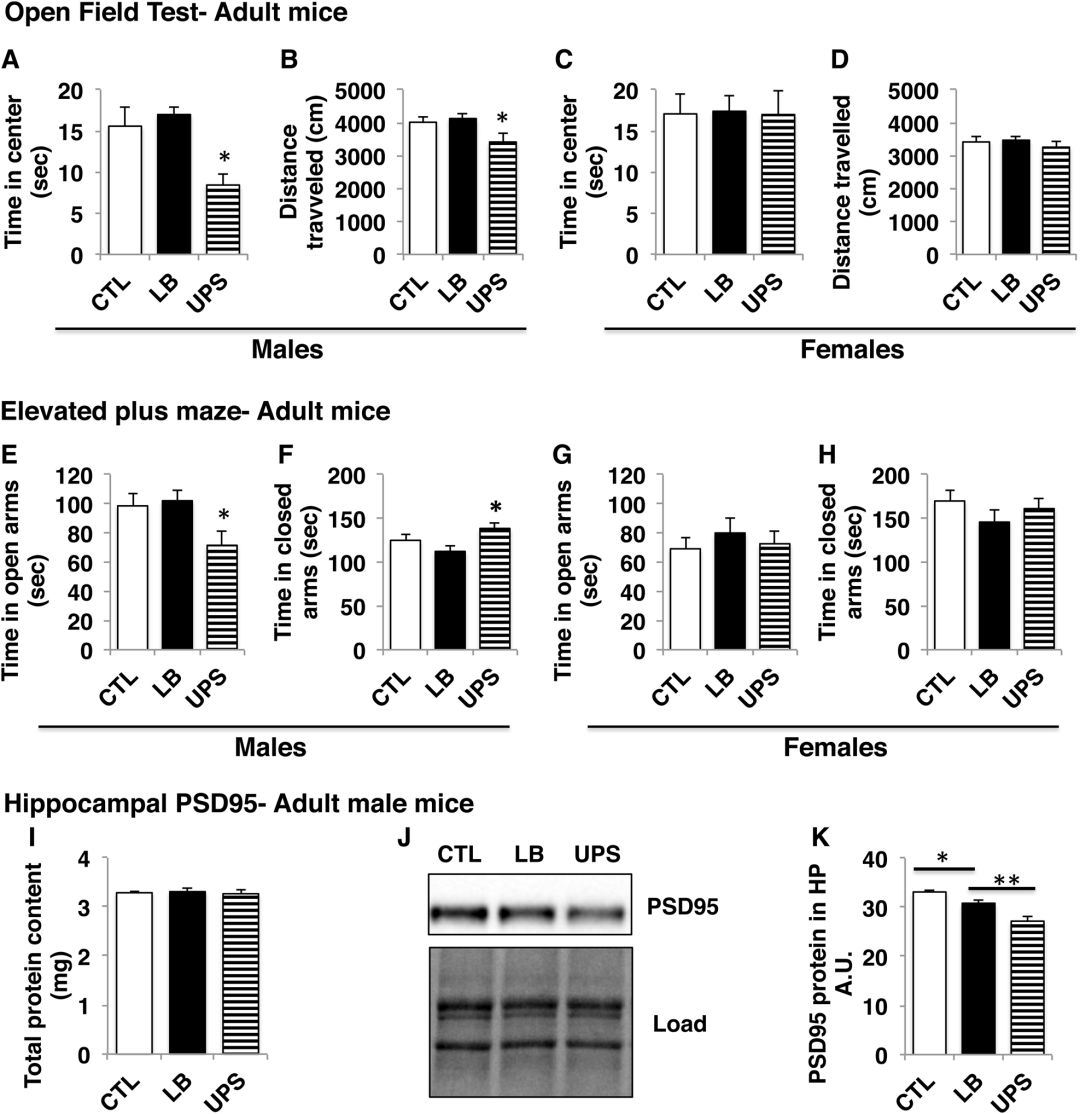
UPS but not LB causes a robust increase in anxiety-like behavior in adult male mice (P70–80). Open-field test (**a**–**d**). Exposure to UPS reduced time spent in the center (**a**) and distance travelled (**b**) in male UPS mice compared to LB and CTL groups. There were no differences between the groups in time spent in the center (**c**) or distance travelled (**d**) in adult females. Elevated plus maze (**e**–**h**). UPS male mice spent less time exploring the open arms (**e**) and more time in the closed arms (**f**) compared to LB and CTL groups. There were no differences between the groups in time spent in the open arms (**g**) or closed arms (**h**) in adult females. Males: CTL = 12–14, LB = 12, UPS = 9–10, Females: CTL = 12, LB = 9–12, UPS = 10. Error bars represent mean ± s.e.m. *p < 0.05, UPS compared to LB and CTL, Tukey-HSD (**a**–**h**). (**i**) Total protein extracted from the hippocampus of adult male mice. (**j**–**k**) Western blot showing levels of PSD95 in the hippocampus of adult male mice. CTL = 5, LB = 5, UPS = 4 (**i**–**k**). *A.U.* arbitrary units. Error bars represent mean ± s.e.m. *p < 0.05, LB compared to CTL and **p < 0.01 UPS compared to LB, Tukey-HSD (**k**)

To determine whether the increase in anxiety seen in LB and UPS juvenile mice persisted in adulthood we tested the effects of ELS and sex on exploration in the open field using a second cohort of adult mice (P70–80, Fig. 1a). There was a significant effect of sex on behavior in the open field (Table S2) prompting us to conduct separate analyses for males and females. In males, there was a significant effect of ELS on time spent in the center of the open field (*F*[2,33] = 5.95, *p* = 0.006) that was due to reduced exploration of the center in UPS compared to CTL (*p* = 0.02) and LB (*p* = 0.007), with no difference between CTL and LB (Fig. 3a). There was also a significant effect of ELS on the distance traveled (*F*[2,33] = 3.76, *p* = 0.034) due to reduced distance travelled in UPS compared to LB (*p* = 0.039) and a trend when compared to CTL (*p* = 0.079), with no difference between LB and CTL (Fig. 3b). In females, ELS had no effect on time in the center (Fig. 3c) or distance travelled (Fig. 3d). Together with our work in juvenile mice, these findings suggest that exposure to LB is associated with increased anxiety in the open field in juvenile but not adult male mice whereas UPS leads to increased anxiety in both juvenile and adult male mice.

Separate analyses for males and females were also conducted for the EPM due to significant effects of sex on time exploring the open and closed arms (Table S2). In males, there was a significant effect of ELS on time exploring the open arms (*F*[2,30] = 3.76, *p* = 0.035) that was due to reduced time in the open arms in UPS mice compared to LB (*p* = 0.04) and a trend when compared to CTL (*p* = 0.076). There was no difference between CTL and LB in time spent in the open arms (Fig. 3e). The effect of ELS on time exploring the closed arms was also significant (*F*[2,30] = 3.67, *p* = 0.038) with post hoc analysis indicating significant increase in the UPS compared to LB (*p* = 0.029), but no statistical differences between CTL and LB (Fig. 3f). Similar to the open-field data, ELS did not affect behavior in the EPM in adult females (Fig. 3g, h). ELS did not affect total distance travelled in the elevated plus maze in adult males or females (Fig S2).

Together, these findings demonstrate that exposure to UPS, but not LB, causes a robust increase in anxiety-like behavior in adult male mice. These observations prompted as to examine whether UPS causes also more pronounced abnormalities in the expression of the synaptic marker PSD95 in the hippocampus of adult male mice (CTL: *n* = 5, LB: *n* = 5, UPS: *n* = 4). The total proteins content in the hippocampus were similar between CTL, LB, and UPS (Fig. 3i), but there was a significant effect of ELS on PSD95 (*F*[2,11] = 22.43, *p* < 0.0005, one-way ANOVA, Fig. 3j, k) that was due to reduced levels of LB compared CTL (*p* = 0.047) and further reduction in UPS compared to LB (*p* = 0.005). These findings demonstrate that the effects of LB on levels of PSD95 in the hippocampus persist in adulthood (see also Fig. 1e, f) and suggest a more severe long-term synaptic changes in UPS male mice compared to LB and CTL mice.

### UPS is associated with amygdala hyperconnectivity with the prefrontal cortex (PFC) and the hippocampus (HPC)

Next, we used rsfMRI to compare the connectivity between the amygdala, PFC, dorsal hippocampus (dHPC), ventral hippocampus (vHPC), and the hypothalamus in adult CTL and UPS male mice (CTL: *n* = 5, UPS: *n* = 6). This setup creates a network of 10 nodes (5 nodes in each hemisphere) that are linked via 45 unique pairs of connections (Fig S3). These brain regions were selected because of their role in modulating anxiety-like behavior in the mouse^57–59^ and because multiple rsfMRI studies have found abnormal connectivity between these brain regions in individuals exposed to childhood maltreatment (Table S1).

There were no differences between the two groups in global brain signal, heart rate, respiratory rate, temperature, amount of urethane used to anesthetize for imaging, or body weight (Table S3). Eleven connections were significantly different between the two groups (*p* < 0.05, two tails) and had large effect sizes (Cohn’s *d* = 1.39–2.02). Four connections were still significant after adjusting for multiple comparisons, all linked to the left amygdala (L-amygdala-L-PFC, L-amygdala-R-PFC, l-amygdala-R-dHPC, l-amygdala-R-vHPC, Table 1). Nine of the 12 connections between the amygdala and the PFC and the amygdala and the HPC were significantly greater in UPS and an additional connection between the right amygdala and the left ventral hippocampus showed a similar trend (*p* = 0.07, Table 1). These observations suggest that exposure to UPS specifically perturbs amygdala connectivity with the HPC and the PFC. Interestingly, connectivity between the amygdala and the hypothalamus was not significantly affected, suggesting that UPS does not equally impact all amygdala connections (Table 1).

**Table 1:** UPS is associated with hyperconnectivity between the amygdala and the prefrontal cortex and the amygdala and the hippocampus. L—left, R—right, amygdala (amyg), Prefrontal cortex (pfc), dorsal hippocampus (dhpc), ventral hippocampus (vhpc), and hypothalamus (hypoth)

Brain region	Control	UPS	Pair-node connections	*P* values	Effect size (*d*)
Amygdala–prefrontal cortex	−0.83	2.34	Lamyg_Lpfc	**0.02** ^*^	1.73
	−0.30	3.05	Lamyg_Rpfc	**0.009** ^*^	2.02
	0.06	1.82	Ramyg_Lpfc	0.15	0.96
	0.25	2.84	Ramyg_Rpfc	**0.03**	1.55
Amygdala–hippocampus	0.80	4.51	Lamyg_Ldhpc	**0.04**	1.44
	0.94	4.15	Lamyg_Rdhpc	0.03*	1.64
	2.35	6.03	Lamyg_Lvhpc	**0.05**	1.40
	1.76	4.39	Lamyg_Rvhpc	**0.02** ^*^	1.72
	0.92	4.08	Ramyg_Lvhpc	0.07	0.92
	3.01	5.03	Ramyg_Rvhpc	0.13	1.05
	0.97	4.27	Ramyg_Ldhpc	**0.04**	1.50
	1.15	3.89	Ramyg_Rdhpc	**0.05**	1.39
Amygdala–hypothalamus	5.62	6.78	Lamyg_Lhypoth	0.64	0.29
	3.64	5.28	Lamyg_Rhypoth	0.58	0.35
	1.80	5.99	Ramyg_Lhypoth	0.19	0.89
	3.15	7.84	Ramyg_Rhypoth	0.15	0.99
Prefrontal cortex–hippocampus	1.81	5.61	Lpfc_Lvhpc	0.34	0.60
	2.67	6.21	Lpfc_Rvhpc	0.44	0.52
	2.22	6.42	Lpfc_Ldhpc	0.32	0.63
	2.20	6.04	Lpfc_Rdhpc	0.35	0.59
	1.71	7.96	Rpfc_Ldhpc	0.14	0.82
	2.35	7.54	Rpfc_Rdhpc	0.23	0.78
	1.88	6.21	Rpfc_Lvhpc	0.30	0.67
	2.36	6.59	Rpfc_Rvhpc	0.30	0.67
Prefrontal cortex–hypothalamus	−0.22	2.76	Lpfc_Lhypoth	0.11	1.12
	0.37	2.93	Lpfc_Rhypoth	0.21	0.85
	−0.49	3.48	Rpfc_Lhypoth	**0.03**	1.59
	0.25	3.76	Rpfc_Rhypoth	0.06	1.36
Hypothalamus–hippocampus	2.68	5.15	Lhypoth_Lvhpc	0.17	0.93
	2.63	4.73	Lhypoth_Rvhpc	0.17	0.95
	1.66	5.29	Lhypoth_Ldhpc	**0.06**	1.36
	1.63	4.77	Lhypoth_Rdhpc	0.08	1.25
	2.09	4.38	Rhypoth_Lvhpc	0.26	0.75
	2.66	4.38	Rhypoth_Rvhpc	0.28	0.72
	1.00	4.63	Rhypoth_Ldhpc	0.08	1.25
	1.23	4.53	Rhypoth_Rdhpc	**0.04**	1.53
Interhemispheric	2.60	5.78	Ramyg_Lamyg	0.31	0.68
	9.65	16.03	Rpfc_Lpfc	0.10	1.09
	9.77	15.87	Rvhpc_Ldhpc	0.13	1.05
	9.42	15.26	Rdhpc_Lvhpc	0.11	1.13
	10.14	18.27	Rvhpc_Lvhpc	0.13	1.05
	12.65	21.17	Rdhpc_Ldhpc	0.15	0.98
	12.38	17.03	Rhypoth_Lhypoth	0.30	0.66
Intrahemispheric	16.34	22.22	Rdhpc_Rvhpc	0.11	1.14
	17.36	22.39	Ldhpc_Lvhpc	0.18	0.92

*P*<0.05 are shown in bold. *Significant after adjusting for multiple comparisons using the sequential goodness of fit

### Amygdala connectivity with the hippocampus and the prefrontal cortex correlates with anxiety-like behavior

**Fig. 4:**
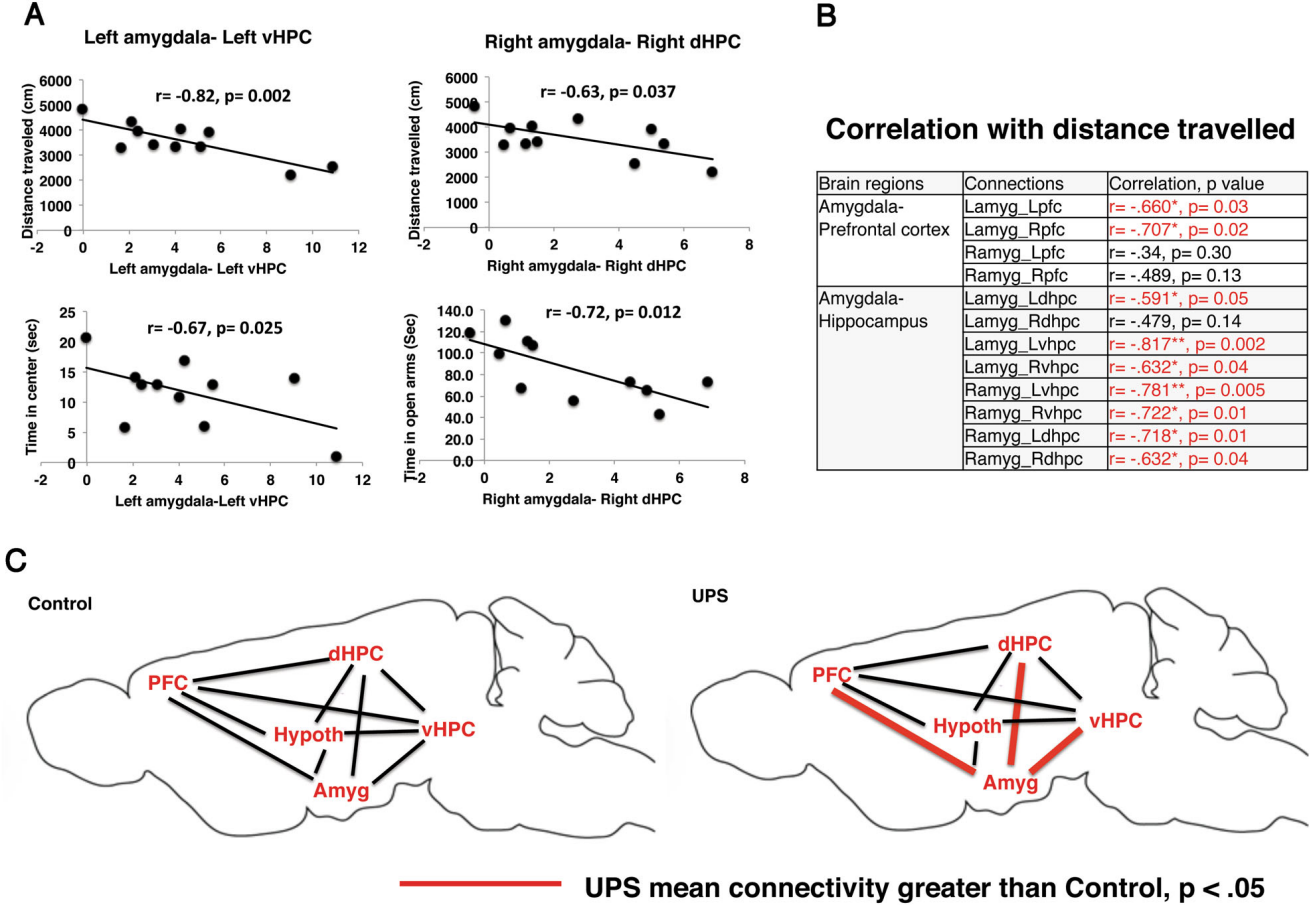
Strength of amygdala–hippocampus and amygdala–prefrontal cortex connectivity is significantly correlated with anxiety-like behavior. **a** The left amygdala-left ventral hippocampus (vHP) connectivity is correlated with the distance travelled (top left) and time in the center (bottom left) in the open field. The right amygdala-right dorsal hippocampus (dHP) connectivity is correlated with distance travelled in the open field (top right) and time spent in the open arms in the elevated plus maze (bottom left). **b** Amygdala connectivity with the PFC and the HPC is highly correlated with distance travelled in the open field. **c** Exposure to UPS increases amygdala-PFC and amygdala-HPC connectivity. Amygdala (Amyg), Prefrontal cortex (PFC), dorsal hippocampus (dHPC), ventral hippocampus (vHPC), and hypothalamus (Hypoth)

To better understand the relationship between our rsfMRI data and our behavioral findings, we used a within-subject analysis to examine the correlation between the strength of the amygdala-PFC and amygdala-HPC connectivity as determined by the normalized Pearson coefficient (see Methods section and Table 1) and anxiety-like behavior in the open field and EPM. We found extensive correlations between frontolimbic connectivity strength and anxiety-like behavior. In Fig. 4a we present functional correlations between the left amygdala-left vHP and the right amygdala-right dHP and anxiety-like behavior in the open field and EPM. The strength of the 12 functional connections between the amygdala and the PFC or the HPC was highly correlated with the total distance travelled in the open field (Fig. 4b).

## Discussion

This is the first study to use rsfMRI to characterize limbic network connectivity in a mouse model of ELS. Using this approach, we found hyperconnectivity between the amygdala and the HPC (both ventral and dorsal) and between the amygdala and the PFC in UPS male mice (for a schematic summary see Fig. 4c). The large effect size of these differences and their presence in both the right and left hemispheres (Table 1) suggest a robust effect of UPS. These findings highlight abnormal amygdala connectivity as an important hub in the miswiring grid of UPS mice, a finding that is consistent with a large body of rsfMRI data in humans exposed to childhood maltreatment^26–32^. In our study, connectivity between the amygdala and the hypothalamus was not significantly affected, suggesting that UPS does not equally perturb all connections to the amygdala. This preferential effect might be due to the fact that connectivity between the amygdala and the hypothalamus is established at P14^60^, prior to the onset of the unpredictable stress in the UPS paradigm (see Fig. 1a for timeline). The strength of the connectivity between the amygdala and the HPC and the amygdala and PFC was highly correlated with anxiety-like behavior (Fig. 4a, b) suggesting that hyperconnectivity in the limbic network supports the increase in anxiety-like behavior seen in UPS male mice. This finding is consistent with work showing that reduced anxiety and stress reactivity seen in Ahi1 heterozygous knockout mice is associated with reduced rsfMRI connectivity between the amygdala and several other brain regions including the vHPC^61^. Moreover, optogenetic activation of amygdala afferents to the PFC^58^ or the vHPC^59^ have been shown to increase anxiety, while inhibiting these connections has anxiolytic effects in the open field and the EPM.

Another important finding reported here is that UPS, but not LB, leads to robust increase in anxiety-like behavior in male mice. LB juvenile male mice showed increased anxiety in the open field (Fig. 2a) but not in the EPM (Fig. 2d, e). Exposure to LB had no effect on behavior in the open field or the EPM in adulthood (Fig. 3). These findings are consistent with previous work showing no increase in anxiety-like behavior^41,62^ or inconsistent results^63^ in the open field test and EPM in adult mice and rats exposed to LB. In contrast, exposure to UPS increased anxiety-like behavior in the open field and EPM in both juvenile and adulthood (Figs. 2–3). These results mirror clinical studies showing a dose–response relationship between the number of adverse events experienced early in life and the risk for multiple psychopathologies, including anxiety^1,6^. Exactly how multiple stressors early in life synergize to alter neurodevelopment is an open question that has not received much attention in animal models of ELS. Moreover, most of the ELS paradigms currently available in rodents rely on predictable and homotypic stressors such as daily maternal separation, low levels of maternal care, or limited bedding^33,34,64^. Our findings suggest that exposure to LB causes developmental abnormalities (Fig. 1) that sensitize the postnatal brain to additional unpredictable threats associated with the UPS paradigm. This notion is best illustrated by the cumulative effect of UPS on levels of the synaptic marker PSD95 in the adult hippocampus (Fig. 3j, k). Future studies will test whether unpredictable maternal separation and nest disruption, in mice raised with normal levels of bedding and nesting material, is sufficient to alter anxiety, stunt growth, or reduce PSD95 levels in the hippocampus. We predict that this is not going to be the case because exposure to prolonged daily maternal separation after the first week of life (P7–20) did not affect body weight or increase anxiety-like behavior in adult rats^65^.

Exposure to LB is sufficient to cause multiple developmental abnormalities including increased anxiety in juvenile males (Fig. 2a), stunted growth (Fig. 1b and Fig S1), abnormal microglial density and morphology (Fig. 1h−k), and reduced expression of the synaptic marker PSD95 in the hippocampus (Figs 1f and 3k). These findings replicate previous findings reported in C57 mice and in rats^17,41,63,66^ indicating that these are not specific for the Balbc/Byj strain or to our modified LB model. The observation that juvenile LB mice show increased locomotor activity in the open-field test (Fig. 2c) is interesting and is consistent with clinical studies showing that exposure to neglect and impoverished conditions early in life increase the risk for attention deficit hyperactivity disorder^7,11,67^.

The effects of amygdala connectivity between the PFC and the HPC in adult LB male mice are yet to be characterized. However, based on the normal anxiety-like behavior seen in adult LB mice we predict more subtle perturbations in frontolimbic connectivity in these mice. This prediction is consistent with recent rsFMRI work examining this issue in juvenile and adult rats exposed to limited bedding from P8–12. In fact, of the nine connections tested between three sub-regions of the amygdala (e.g., basolateral, lateral, central) and three sub-regions of the PFC (e.g., infra-limbic, pre-limbic, anterior cingulate cortex) only one connection (lateral amygdala to anterior cingulate cortex) was significantly reduced in adult rats exposed to limited bedding, an effect that did not survive correction for multiple comparisons^68^.

The observation that males are more sensitive than females to the behavioral effects of UPS is consistent with findings from several other groups (reviewed in ref. 69), but the mechanisms responsible for this sex-specific effects are yet to be elucidated in rodent models of postnatal stress. Future studies will assess network connectivity in UPS female mice. These studies will determine whether UPS causes sex-specific hyperconnectivity in males and may help clarify conflicting results in humans about the moderating effects of sex on frontolimbic connectivity (Table S1) and risk for psychopathology in individuals exposed to childhood asdersity^8–14^.

Abnormal frontolimbic connectivity seen with rsfMRI in adult UPS male mice might be due to alterations in direct monosynaptic connections between the affected brain regions. This assertion is supported by optogenetic work showing that direct monosynaptic connections between the amygdala and the vHPC^59^ and between the amygdala and the PFC^58^ regulate anxiety-like behavior in the open-field test and the EPM. In addition, alterations in rsfMRI are in many cases linked to structural changes that can be detected using high-resolution diffusion tensor imaging^70,71^. Such imaging-based tractography coupled with tracing and optogenetics work can help identify structural changes in the limbic network of UPS mice and clarify their contribution to anxiety-like behavior.

The hyperconnectivity seen in UPS male mice might be due to impaired synaptic or dendritic pruning^55^. A normally developing brain initially forms an exuberant and overly connected grid that is pruned by microglia in an activity-dependent manner. A failure to remove redundant and non-functional synapses during a critical period of development may leave behind an abnormally hyperconnected limbic network^55^. In support of this assertion we show that exposure to LB reduces microglial density and morphological complexity in the hippocampus of 14-day-old pups (Fig. 1h−k). This finding replicates previous work in C57 mice^66^ and is important because the hippocampus undergoes intense synaptic pruning during this critical developmental period^72,73^. Moreover, we have preliminary data showing that UPS causes more significant alterations in microglial gene expression compared to LB male mice^55,74^. Although most pruning activity occurs early in life, there is evidence that almost 50% of the connections between the PFC and the amygdala are pruned as rats transition from adolescence (P45) to adulthood (P90)^75^. Inhibition of this pruning process in UPS male mice may explain the hyperconnectivity between these two brain regions in adult UPS mice. Interestingly, a recent longitudinal rsFMRI imaging study using a large cohort of normally developing children found a reduction in functional and structural connectivity between the amygdala and the PFC during the transition from adolescence to adulthood^76^. Importantly, increased amygdala-PFC connectivity was associated with increased anxiety and depression^76^. These findings raise the possibility that UPS disrupts this normal refinement process resulting in the retention of ‘adolescent-like’ hyperconnectivity and elevated levels of anxiety in adult UPS male mice.

### Concluding remarks

Our findings make two important contributions to previous work examining the effects of ELS on anxiety-like behavior in the mouse. First, we show that UPS, but not LB, leads to robust increase in anxiety-like behavior in male mice, indicating that complex and unpredictable stressors early in life lead to more significant perturbation in the circuitry that regulates anxiety-like behavior. This is an important finding given that most of the ELS paradigms currently available in rodents rely on predictable and homotypic stressors. Second, this work is the first to link hyperconnectivity in frontolimbic circuits with increased anxiety in a mouse model of ELS allowing for more mechanistic understanding of parallel findings in humans^30–32^.

## Electronic supplementary material

Supplemental information
