# Supplementary material for: Amygdala hyper-connectivity in a mouse model of unpredictable early life stress
Source: Transl Psychiatry. 2018 Feb 21;8:49. doi: 10.1038/s41398-018-0092-z (PMC5820270; doi:10.1038/s41398-018-0092-z)
Supplement: Supplementary file 1 — Supplemental information [file 41398_2018_92_MOESM1_ESM.pdf]

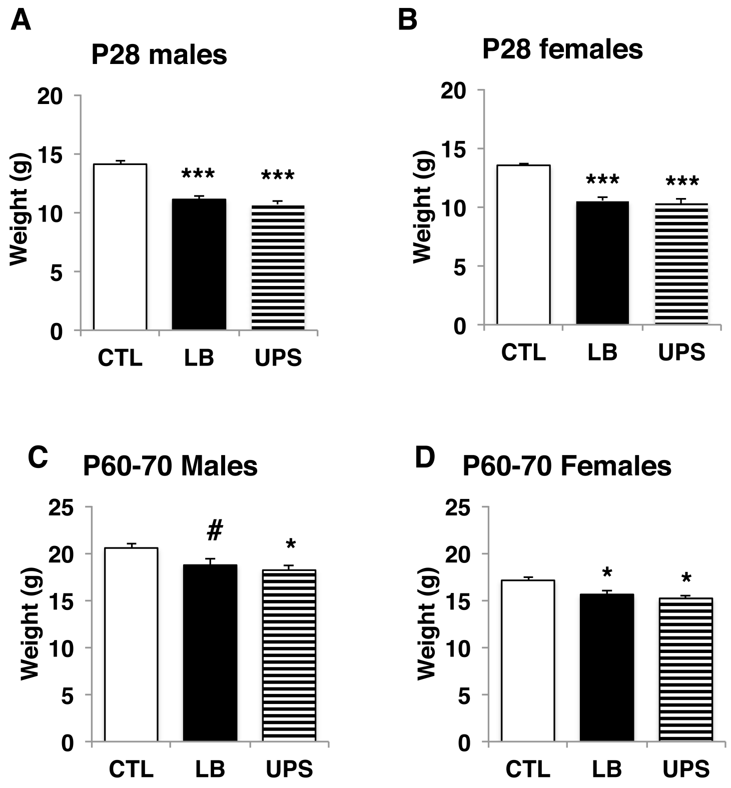

Fig S1. Effects of LB and UPS on body weight in P28 males (A) and females (B). Males: CTL= 34, LB= 25, UPS= 25. Females: CTL= 24, LB= 32, UPS= 33. Similar pattern was seen in young adult male (C) and female mice (D). There were no differences between LB and UPS at these ages. Males: CTL= 12, LB= 12, UPS= 12. Females: CTL= 12, LB= 10, UPS= 10. #p= 0.067, *p< 0.05, ***p< 0.0005 compared to CTL group, Tukey-HSD.

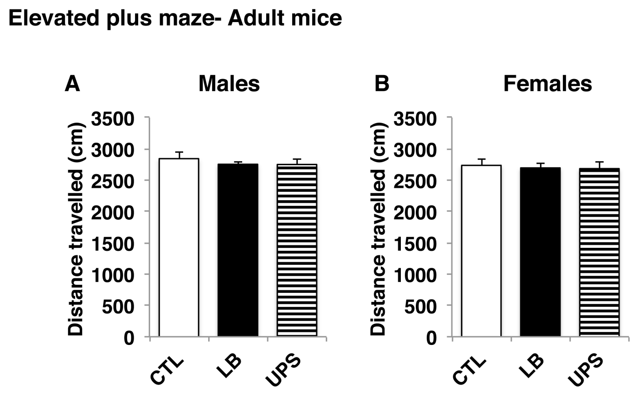

Fig S2. There were no differences between the groups in total distance travelled in the EPM for males (A) or female adult mice (B). Males: CTL= 12, LB= 12, UPS =9, Females CTL= 12, LB= 9, UPS= 10. Error bars represent mean ± SEM.

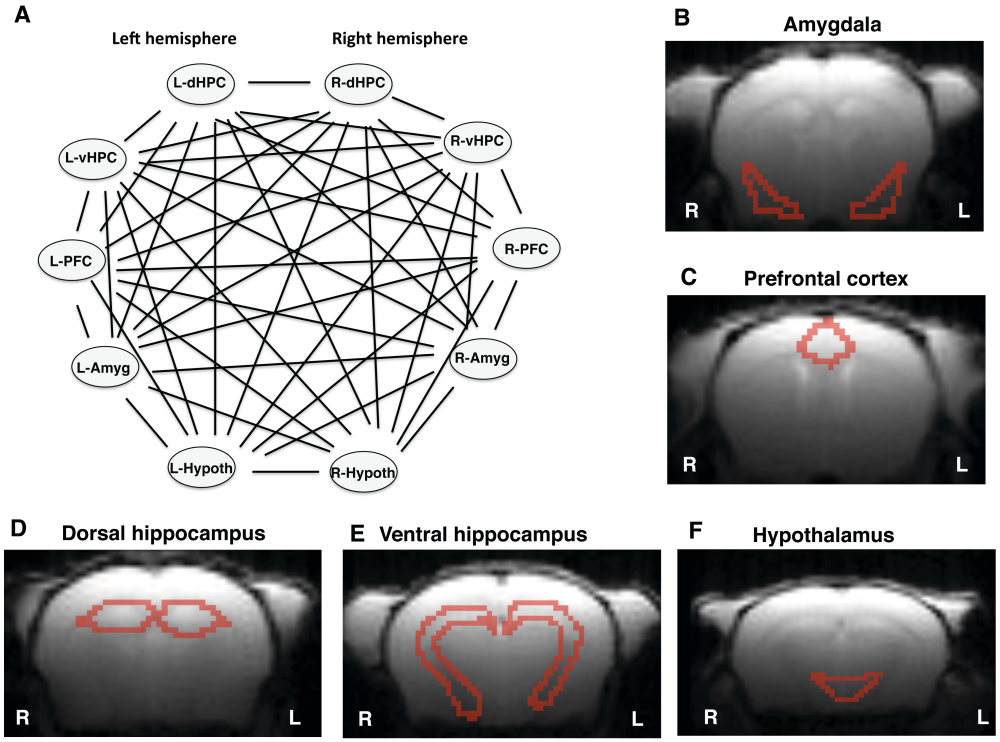

Fig S3. Resting state FMRI setup. (A) Schematic diagram of the connectivity grid between the amygdala (Amyg), prefrontal cortex (PFC), dorsal hippocampus (dHPC), ventral hippocampus (vHPC), and hypothalamus (Hypoth). Region of interest maps for the amygdala (B), prefrontal cortex (C), dorsal hippocampus (D), ventral hippocampus (E), and hypothalamus (F).

Table S1. Summary of clinical literature examining the effects of childhood maltreatment on rsfMRI connectivity between the amygdala, prefrontal cortex, and the hippocampus. Abbreviations: CEM-childhood emotional abuse, CTQ- childhood trauma questionnaire, dACC- dorsal anterior cingulate cortex, dmPFC- dorsal medial prefrontal cortex, ELS-early life stress, DN- default network, HPC- hippocampus, NEMESIS- Netherlands Mental Health Survey and Incidence Study trauma, PCC- posterior cingulate cortex, sgACC- subgenual anterior cingulate cortex, vlPFC- ventrolateral prefrontal cortex, vmPFC- ventral medial prefrontal cortex.

Publication	Form of childhood maltreatment	Subjects	Amygdala-PFC	Amygdala-HPC	HPC-PFC	Other findings
**Van der werff 2013**[1]	Childhood Emotional abuse (CEM)	Controls: n=44. CEM n=44. 50% females, ages 37-38.Used the NEMESIS trauma interview to assess CEM. No differences in depression or anxiety between the groups.	No difference	Reduced connectivity between the left Amygdala and the right hemisphere including the right HP, no effect in the left amygdala	No difference	CEM was associated with reduced connectivity between the dACC and the precuneus and also frontal regions of the brain. No effect of CEM on default mode network.
**Birn 2014**[2]	Mild, most CTQ 25-40	27 veterans with significant combat experience, several of which met criteria for traumatic brain injury, all males, ages 27, 17/27 met criteria for PTSD, CTQ was between 25-65, but only 4 subjects with CTQ > 45	High CTQ score was associated with reduced connectivity between the PFC (dmPFC and vmPFC) and the amygdala	No difference	High CTQ score was associated with reduced connectivity between the HPC and mPFC	PTSD is associated with increased connectivity between the amygdala and the dorsal PFC (dlPFC and dmPFC)

**Burghy 2012**[3]	Maternal stress (similar cohort to the one used in Herringa 2013)	57 adolescents, mean age 18, half females.	No direct effect of maternal stress on connectivity (reduced connectivity is mediated via elevated cortisol levels at age 4 in females only.	No direct effect of maternal stress on connectivity (reduced connectivity is mediated via elevated cortisol levels at age 4 in females only	Not assessed	Using structural equation model they found that cortisol at age 4 was associated with reduced amygdala-vmpfc connectivity which in turn was inversely correlated with anxiety and positively correlated with depression in females but not males.
**Herringa 2013**[4]	Mild, most CTQ 25-40	64 adolescents, 30 females, mean age 18. Also collected data for internalizing symptoms.	Reduced connectivity between R-amyg and vmPFC (mainly the sgACC) in females, but not males	Not assessed	Reduced connectivity between the L-HPC and the sgACC in both males and females	In females, reduced connectivity between the sgACC and both the amygdala and the L-HPC were associated with increase internalizing symptoms while in males the reduced connectivity between the L-HPC and the sg-ACC was associated with increased internalizing symptoms.

**Dean 2014**[5]	Moderate, CTQ=45	15 adults, 8 men, with amphetamine use disorder, age 38. These were drug free for several days before undergoing assessment and rsfMRI study. This cohort scored high on the CTQ indicating positive ELS (CTQ= 45), but with no depression or anxiety.	High CTQ was associated with increased connectivity between amygdala and orbitofrontal cortex	Positive correlation between CTQ and amygdala-HPC connectivity	Not assessed	The connectivity between the amygdala and the right HPC was positively correlated with depression, anxiety and self-regulation and negatively correlated with mindfulness and self compassion.
**Philip 2013**[6]	Moderate-severe. Used subclasses of CTQ scores	ELS n=13 (moderate to severe) and CTL n=9 individuals, half females, mean ages 36. No differences in psychopathology between ELS and CTL	High CTQ was associated with a trend (p= 0.07) for increased connectivity between the amygdala and the mPFC in ELS	Not assessed	Not assessed	Reduced connectivity in the DN (PCC and mPFC)
**Cisler 2013**[7]	Moderate, CTQ 27-47, adult woman	Used 3 groups: 1) woman with no ELS and no depression, ages 26, CTQ 27, n=12, CTL 2) woman with ELS but no depression, ages 27, CTQ 47, n=7, “resilient”, 3) woman with ELS and depression, ages 31, CTQ 67, n=17, “susceptible”.	Reduced in betweenness centrality for vlPFC in resilient individuals compared to both groups.	Increased betweenness centrality for amygdala in susceptible compared to resilient	Reduced vlPFC in betweenness centrality in resilient individuals compared to both groups	Using graph theory they found that Increased hub like connectivity of the amygdala is associated with susceptibility while reduced PFC connectivity is associated with resiliency.

**Wang 2014**[8]	Emotional and physical neglect	Used three groups: 1) MDD without childhood neglect (n=20, HAMD=27, 2) MDD with childhood neglect (n=18, used only emotional and physical neglect, CTQ-EN=15, CTQ-PN=11, HAMD=27, 3) healthy controls (n=20), about half were females, ages 28	Using whole brain connectivity they found reduced connectivity in the PFC, HPC, and amygdala in individuals with MDD and a history of childhood neglect compared to MDD without childhood neglect	Using whole brain connectivity they found reduced connectivity in the PFC, HPC, and amygdala in individuals with MDD and a history of childhood neglect compared to MDD without childhood neglect	Using whole brain connectivity they found reduced connectivity in the PFC, HPC, and amygdala in individuals with MDD and a history of childhood neglect compared to MDD without childhood neglect	

Table S2. Statistical summary of the Behavioral testing.

Juvenile mice	Ages	Number of mice	ELS	Sex	Interaction	Outcome
Open field-Time in the center	P35-40	CTL / LB / UPS<br>M: 12 / 12 / 11<br>F: 14 / 9 / 14	F(2,32)= 6.10, **p= 0.006** (males)<br>F(2,34)= 0.32, p= 0.73 (females)	F(1,66)= 1.018, p= 0.28	F(2,66)= 4.81, **p= 0.011**	Reduced in LB and UPS males.
Open field-Distance travelled	P35-40	CTL / LB / UPS<br>M: 12 / 12 / 11<br>F: 14 / 9 / 14	F(2,66)= 8.33, **p= 0.001**	F(1,66)= 1.07, p= 0.30	F(2,66)= 0.14, p= 0.87	Increased in LB males and females
EPM-Time in open arms	P35-40	CTL / LB / UPS<br>M: 12 / 12 / 10<br>F: 13 / 9 / 12	F(2,62)= 4.53, **p= 0.015**	F(1,62)= 0.54, p= 0.46	F(2,62)= 0.83, p= 0.44	Reduced in UPS males and females
EPM-Time in closed arms	P35-40	CTL / LB / UPS<br>M: 12 / 12 / 10<br>F: 13 / 9 / 12	F(2,62)= 8.21, **p= 0.001**	F(1,62)= 0.08, p= 0.78	F(2,62)= 0.18, p= 0.83	Increased in UPS males and females
**Adult mice**	**Ages**	**Number of mice**	**ELS**	**Sex**	**Interaction**	**Outcome**
Open field-Time in the center	P70-85	CTL / LB / UPS<br>M: 14 / 12 / 10<br>F: 12 / 12 / 10	F(2,33)= 5.95, **p= 0.006** (males)<br>F(2,31)= 0.007, p= 0.99 (females)	F (1,64)= 4.00, **p= 0.05**	F (2,64)= 2.01, p= 0.14	Reduced in UPS males
Open field-Distance travelled	P70-85	CTL / LB / UPS<br>M: 14 / 12 / 10<br>F: 12 / 12 / 10	F(2,33)= 3.76, **p= 0.034** (males)<br>F(2,31)= 0.55, p= 0.58 (females)	F (1,64)= 11.61, **p= 0.001**	F (2,64)= 1.22, p= 0.30	Reduced in UPS males
EPM-Time in open arms	P70-85	CTL / LB / UPS<br>M: 12 / 12 / 9<br>F: 12 / 9 / 10	F (2,30)= 3.76, **p= 0.035** (males)<br>F (2,28)= 0.42, p= 0.66 (females)	F(1,58)= 6.07, **p= 0.017**	F(2,58)= 1.76 p= 0.18	Reduced in UPS males

EPM-Time in closed arms	P70-85	CTL / LB / UPS<br>M: 12 / 12 / 9<br>F: 12 / 9 / 10	F (2,30)= 3.67, **p= 0.038** (males)<br>F (2,28)= 0.98, p= 0.39 (females)	F(1,58)= 18, **p< 0.0005**	F(2,58)= 0.67 p= 0.51	Increased in UPS males
EPM-distance travelled	P70-85	CTL / LB / UPS<br>M: 12 / 12 / 9<br>F: 12 / 9 / 10	F (2,30)= 0.52, p= 0.6 (males)<br>F (2,28)= 0.08, p= 0.92 (females)	F(1,58)= 1.15 p= 0.23	F(2,58)= 0.05, p= 0.95	No change

Table S3. There were no differences in the average global signal, heart rate, respiratory rate, temp, urethane used, and weight between the CTL and UPS mice used for imaging.

	Group	Sample size	Mean	SEM	P values
Global brain signal (BOLD)	CTL	5	-0.00258	0.000411	0.43
	UPS	6	-0.00352	0.000973	
Heart rate (beats/sec)	CTL	5	5.85	0.152	0.72
	UPS	6	5.73	0.264	
Respiratory rate (breaths/sec)	CTL	5	2.89	0.103	0.44
	UPS	6	2.71	0.184	
Temp (C°)	CTL	5	37.38	0.090	0.31
	UPS	6	37.53	0.098	
Urethane used (g/kg)	CTL	5	1.99	0.075	0.68
	UPS	6	1.95	0.060	
Weight (g)	CTL	5	27.6	1.26	0.33
	UPS	6	26.1	0.96	

.

References

1. van der Werff SJ, Pannekoek JN, Veer IM, van Tol MJ, Aleman A, Veltman DJ *et al.* Resting-state functional connectivity in adults with childhood emotional maltreatment. *Psychol Med* 2013; **43**(9)**:** 1825-1836.

2. Birn RM, Patriat R, Phillips ML, Germain A, Herringa RJ. Childhood maltreatment and combat posttraumatic stress differentially predict fear-related fronto-subcortical connectivity. *Depress Anxiety* 2014; **31**(10)**:** 880-892.

3. Burghy CA, Stodola DE, Ruttle PL, Molloy EK, Armstrong JM, Oler JA *et al.* Developmental pathways to amygdala-prefrontal function and internalizing symptoms in adolescence. *Nat Neurosci* 2012; **15**(12)**:** 1736-1741.

4. Herringa RJ, Phillips ML, Fournier JC, Kronhaus DM, Germain A. Childhood and adult trauma both correlate with dorsal anterior cingulate activation to threat in combat veterans. *Psychol Med* 2013; **43**(7)**:** 1533-1542.

5. Dean AC, Kohno M, Hellemann G, London ED. Childhood maltreatment and amygdala connectivity in methamphetamine dependence: a pilot study. *Brain and Behavior* 2014; **4**(6)**:** 867-876.

6. Philip NS, Sweet LH, Tyrka AR, Price LH, Bloom RF, Carpenter LL. Decreased default network connectivity is associated with early life stress in medication-free healthy adults. *European neuropsychopharmacology : the journal of the European College of Neuropsychopharmacology* 2013; **23**(1)**:** 24-32.

7. Cisler JM, James GA, Tripathi S, Mletzko T, Heim C, Hu XP *et al.* Differential functional connectivity within an emotion regulation neural network among individuals resilient and susceptible to the depressogenic effects of early life stress. *Psychological Medicine* 2013; **43**(3)**:** 507-518.

8. Wang L, Dai Z, Peng H, Tan L, Ding Y, He Z *et al.* Overlapping and segregated resting-state functional connectivity in patients with major depressive disorder with and without childhood neglect. *Human brain mapping* 2014; **35**(4)**:** 1154-1166.
